# Supplementary material for: Vaccinia virus mRNAs containing long 5′-poly(A)-leaders lack a canonical 5′-methylguanosine cap
Source: Nat Commun. 2025 Dec 22;16:11340. doi: 10.1038/s41467-025-67916-w (PMC12722222; doi:10.1038/s41467-025-67916-w)
Supplement: Supplementary file 1 — Supplementary information [file 41467_2025_67916_MOESM1_ESM.pdf]

Supplementary Materials for

Vaccinia virus mRNAs containing long 5'-poly(A)-leaders lack a canonical 5'-methylguanosine cap

Václav Vopálenský*, Michal Sýkora, Zora Mělková, Ivan Barvík, Kamila Horáčková, Tomáš Mašek, Martin Pospíšek*

Correspondence to: martin.pospisek@natur.cuni.cz (M.P.); vaclav.vopalensky@natur.cuni.cz (V.V.)

This PDF file includes:

Figs. S1 to S5
Tables S1 to S9
Supplementary references

A5R

114145 ATTGCGATTATAAGATTAAATG 114166

AAAAAAAAAATG
GAAAAAAATG
GAAAAAAATG
AAAAAAATG
AAAAAAATG
GAAAAATG
GAAAAATG
AAAATG
AAAATG
AAAATG
AAAATG
GAAATG
GAAATG
GAAATG
GAAATG
GATG
GATG

D12L

C 108214 ACAAACTATAGAGTTGTAAATG 108193 C

GAAAAAAATG
AAAAAAAATG
AAAAAAATG
AAAAAAATG
AAAAAAATG
GAAAAAATG
AAAAAATG
AAAAAATG
GAAAAATG
AAAAATG
AAAATG
GAAATG
GAAATG
GAAATG
AAATG

D5R

98246 CTTTAGTGAAATTTTAACTTGTGTTCTAAATG 98277

AAAAAAAAAAAAAATG
GAAAAAAAAAAATG
AAAAAAAAAAATG
AAAAAAAAATG
AAAAAAAAATG
AAAAAAAATG
GAAAAAAATG
GAAAAAAATG
GAAAAAAATG
GAAAAAAATG
GAAAAAATG
GAAAAAATG
GAAAAAATG
AAAAAAATG
AAAAAAATG
GAAAAATG
AAAAATG
AAAAATG
GAAAATG
GAAAATG
GAAAATG

J6R (WR)

83286 TATTATTTTTATAGTTGTAATA 83307

```
**G**ATAGTTGTAATA
**G**ATAGTTGTAATA
**G**ATAGTTGTAATA
**G**ATAGTTGTAATA
**G**ATAGTTGTAATA
**G**ATAGTTGTAATA
  **G**AGTTGTAATA
  **G**AGTTGTAATA
  **G**AGTTGTAATA
  **G**AGTTGTAATA
  **G**AGTTGTAATA
  **G**AGTTGTAATA
  **G**AGTTGTAATA
  **G**AGTTGTAATA
  **G**AGTTGTAATA
  **G**AGTTGTAATA
  **G**AGTTGTAATA
  **G**AGTTGTAATA
  **G**AGTTGTAATA
   **G**GTTGTAATA
```

J6R (vD9muD10mu)

83449 AAAGAAACTGACTTGATAAAAATG 83472

```
AAAAAAAAAAATG
 AAAAAAAAAATG
 AAAAAAAAAATG
  AAAAAAAAATG
  **G**AAAAAAAATG
   AAAAAAAATG
   AAAAAAAATG
   AAAAAAAATG
    AAAAAAATG
    AAAAAAATG
    AAAAAAATG
    AAAAAAATG
    AAAAAAATG
    **G**AAAAAATG
    **G**AAAAAATG
     AAAAAATG
     AAAAAATG
     **G**AAAAATG
      AAAAATG
      AAAAATG
```

K1L (WR)

↓

C 25951 ACACTAATTAGCGTCTCGTTTCAGACATG 25923 C

GGTCTCGTTTCAGACATG
GGTCTCGTTTCAGACATG
GGTCTCGTTTCAGACATG
GGTCTCGTTTCAGACATG
GGTCTCGTTTCAGACATG
GGTCTCGTTTCAGACATG
GGTCTCGTTTCAGACATG
GGTCTCGTTTCAGACATG
GGTCTCGTTTCAGACATG
GGTCTCGTTTCAGACATG
GGTCTCGTTTCAGACATG
GGTCTCGTTTCAGACATG
GGTCTCGTTTCAGACATG
GGTCTCGTTTCAGACATG
GGTCTCGTTTCAGACATG
GGTCTCGTTTCAGACATG
GGTCTCGTTTCAGACATG
GGTCTCGTTTCAGACATG
GGTCTCGTTTCAGACATG
GGTCTCGTTTCCGACATG
GGTCTCGTTTCCGACATG
GGTCTCGTTTCCGACATG
GGTCTCGTTCCAGACATG
GGTCTCGTTCCAGACATG
GGTCTCGTTCCAGACATG

K1L (vD9muD10mu)

↓

C 25951 ACACTAATTAGCGTCTCGTTTCAGACATG 25923 C

GGCGTCTCGTTTCAGACATG
GGCGTCTCGTTTCAGACATG
GGTCTCGTTTCAGACATG
GGTCTCGTTTCAGACATG
GGTCTCGTTTCAGACATG
GGTCTCGTTTCAGACATG
GGTCTCGTTTCAGACATG
GGTCTCGTTTCAGACATG
GGTCTCGTTTCAGACATG
GGTCTCGTTTCAGACATG
GGTCTCGTTTCAGACATG
GGTCTCGTTTCAGACATG
GGTCTCGTTTCAGACATG
GGTCTCGTTTCAGACATG
GGTCTCGTTTCAGACATG
GGTCTCGTTTCAGACATG
GGTCTCGTTTCAGACATG
GGTCTCGTTTCAGACATG
GGTCTCGTTTCAGACATG
GGTCTCGTTTCAGACATG
GGTCTCGTTTCAGACATG
GGTCTCGTTTCAGACATG
GGTCTCGTTTCAGACATG
GGTCTCGTTTCAGACATG
GGTCTCGTTTCAGACATG

H5R

91781 ACAAAGGTTCTTGAGGGTTGTG~68nts~ATG 91873

↓ (TSS before GAGGGTTGTG)

GGAGGGTTGTG~68nts~ATG
GGAGGGTTGTG~68nts~ATG
GGAGGGTTGTG~68nts~ATG
GGAGGGTTGTG~68nts~ATG
GGAGGGTTGTG~68nts~ATG
GGAGGGTTGTG~68nts~ATG
GGAGGGTTGTG~68nts~ATG
GGAGGGTTGTG~68nts~ATG
GGAGGGTTGTG~68nts~ATG
GGAGGGTTGTG~68nts~ATG
GGAGGGTTGTG~68nts~ATG
GGAGGGTTGTG~68nts~ATG
GGAGGGTTGTG~68nts~ATG
GGAGGGTTGTG~68nts~ATG
GGAGGGTTGTG~68nts~ATG
GGAGGGTTGTG~68nts~ATG
GGAGGGTTGTG~68nts~ATG
GGAGGGTTGTG~68nts~ATG
GGAGGGTTGTG~68nts~ATG
GGAGGGTTGTG~68nts~ATG

I4L

C 64267 AATAATTTTTTTATTACACCAA 64246 C

↓ (TSS before ATTACACCAA)

GATTACACCAA
GATTACACCAA
GATTACACCAA
GATTACACCAA
GATTACACCAA
GATTACACCAA
GATTACACCAA
GATTACACCAA
GATTACACCAA
GATTACACCAA
GATTACACCAA
GATTACACCAA
GATTACACCAA
GATTACACCAA
GATTACACCAA
GATTACACCAA
GATTACACCAA
GACACCAA
GACACCAA
GACACCAA
GACACCAA
GACACCAA
GACACCAA

68 nts = TTAAATTGAAAGCGAGAAATAATCATAAATTATTTCATTATCGCGATATCCGTTAAGTTTGTATCGTAATG

Fig. S1. 5' RACE analysis of selected VACV early gene transcripts.
The upper sequence in each panel corresponds to the viral template DNA. The TSS (black arrow) and INR (underlined) were annotated according to [1, 2]. If our TSS annotation differs, it is marked by an orange arrow. The sequences depicted below the template DNA represent individual sequenced cDNA clones. At least 15 independent cDNA clones were sequenced and aligned with the VACV genomic DNA sequence. Nucleotides identical to the viral template DNA are labeled in red. Nucleotides added in a nontemplated manner are labeled in green. The guanosine residues corresponding to the 5' mRNA cap are marked in black. All sequences are shown in the 5'-to-3' orientation, regardless of their transcriptional orientation in the VACV genome.

G8R (WR)

75177 TAAATAATTTACAAAAATTTAAAATG 75202

AAAAAAAAAAAAAAAAAAATG
AAAAAAAAAAAAAAAAATG
AAAAAAAAAATG
AAAAAAAAATG
AAAAAAAATG
AAAAAAAATG
AAAAAAAATG
AAAAAAAATG
AAAAAAATG
AAAAAAATG
AAAAAATG
GAAAAAATG
AAAAAATG
GAAAAATG
GAAAAATG
GAAAAATG
GAAAATG
GAAAATG

G8R (vD9muD10mu)

75177 TAAATAATTTACAAAAATTTAAAATG 75202

AAAAAAAAAAAAAAAAAATG
AAAAAAAAAAAAAAAAAATG
AAAAAAAAAAAAAAATG
AAAAAAAAAAAAAAATG
AAAAAAAAAAAAATG
AAAAAAAAAAAAATG
GAAAAAAAAAAAATG
GAAAAAAAAAAATG
AAAAAAAAAATG
AAAAAAAAAATG
AAAAAAAAAATG
AAAAAAAAAATG
AAAAAAAAATG
AAAAAAAAATG
AAAAAAAATG
AAAAAAAATG
AAAAAAATG
AAAAAAATG
AAAAAAATG
GAAAAAATG
GAAAAATG
GAAAATG

A2L (WT)

C 111153 111050 C

```
CAGCCCAAAAAACTAAAAATATTACAGGATTTTCTGGCAACGTCTAGAAATAAAATGTTTTTATATAAAATATTGGACGACGAGATACGTAGAGTGTTAACATG

AAAAAAAAACTAAAAATATTACAGGATTTTCTGGCAACGTCTAGGAATAAAATGTTTTTATATAAAATATTGGACGACGAGATACGTAGAGTGTTAACATG
GAAAAAAAAAAATATTGGACGACGAGATACGTAGAGTGTTAACATG
AAAAAAAAAAATATTGGACGACGAGATACGTAGAGTGTTAACATG
AAAAAAAAATATTGGACGACGAGATACGTAGAGTGTTAACATG
GAAAAAAAATATTGGACGACGAGATACGTAGAGTGTTAACATG
AAAAAAAATATTGGACGACGAGATACGTAGAGTGTTAACATG
AAAAAAAATATTGGACGACGAGATACGTAGAGTGTTAACATG
AAAAAAAATATTGGACGACGAGATACGTAGAGTGTTAACATG
AAAAAAAATATTGGACGACGAGATACGTAGAGTGTTAACATG
GAAAAAAATATTGGACGACGAGATACGTAGAGTGTTAACATG
GAAAAAAATATTGGACGACGAGATACGTAGAGTGTTAACATG
GAAAAAAATATTGGACGACGAGATACGTAGAGTGTTAACATG
GAAAAAAATATTGGACGACGAGATACGTAGAGTGTTAACATG
GAAAAAAATATTGGACGACGAGATACGTAGAGTGTTAACATG
AAAAAAATATTGGACGACGAGATACGTAGAGTGTTAACATG
AAAAAAATATTGGACGACGAGATACGTAGAGTGTTAACATG
AAAAAATATTGGACGACGAGATACGTAGAGTGTTAACATG
AAAAAATATTGGACGACGAGATACGTAGAGTGTTAACATG
AAAAAATATTGGACGACGAGATACGTAGAGTGTTAACATG
AAAAAATATTGGACGACGAGATACGTAGAGTGTTAACATG
AAAAAATATTGGACGACGAGATACGTAGAGTGTTAACATG
GAAAAATATTGGACGACGAGATACGTAGAGTGTTAACATG
```

A2L (vD9muD10mu)

C 111153 111050 C

```
CAGCCCAAAAAACTAAAAATATTACAGGATTTTCTGGCAACGTCTAGAAATAAAATGTTTTTATATAAAATATTGGACGACGAGATACGTAGAGTGTTAACATG

AAAAAAAAACTAAAAATATTACAGGATTTTCTGGCAACGTCTAGAAATAAAATGTTTTTATATAAAATATTGGACGACGAGATACGTAGAGTGTTAACATG
AAAAAAAAACTAAAAATATTACAGGATTTTCTGGCAACGTCTAGAAATAAAATGTTTTTATATAAAATATTGGACGACGAGATACGTAGAGTGTTAACATG
AAAAAAAAACTAAAAATATTACAGGATTTTCTGGCAACGTCTAGAAATAAAATGTTTTTATATAAAATATTGGACGACGAGATACGTAGAGTGTTAACATG
AAAAAAAAACTAAAAATATTACAGGATTTTCTGGCAACGTCTAGAAATAAAATGTTTTTATATAAAATATTGGACGACGAGATACGTAGAGTGTTAACATG
AAAAAAAAACTAAAAATATTACAGGATTTTCTGGCAACGTCTAGAAATAAAATGTTTTTATATAAAATATTGGACGACGAGATACGTAGAGTGTTAACATG
AAAAAAAAACTAAAAATATTACAGGATTTTCTGGCAACGTCTAGAAATAAAATGTTTTTATATAAAATATTGGACGACGAGATACGTAGAGTGTTAACATG
AAAAAAAAACTAAAAATATTACAGGATTTTCTGGCAACGTCTAGGAATAAAATGTTTTTATATAAAATATTGGACGACGAGATACGTAGAGTGTTAACATG
AAAAAAAAACTAAAAATATTACAGGATTTTCTGGCAACGTCTAGGAATAAAATGTTTTTATATAAAATATTGGACGACGAGATACGTAGAGTGTTAACATG
AAAAAAAAACTAAAAATATTACAGGATTTTCTGGCAACGTCTAGGAATAAAATGTTTTTATATAAAATATTGGACGACGAGATACGTAGAGTGTTAACATG
AAAAAAAAACTAAAAATATTACAGGATTTTCTGGCAACGTCTAGGAATAAAATGTTTTTATATAAAATATTGGACGACGAGATACGTAGAGTGTTAACATG
AAAAAAAAACTAAAAATATTACAGGATTTTCTGGCAGCGTCTAGGAATAAAATGTTTTTATATAAAATATTGGACGACGAGATACGTAGAGTGTTAACATG
AAAAAAAACTAAAAATATTACAGGATTTTCTGGCAACGTCTAGAAATAAAATGTTTTTATATAAAATATTGGACGACGAGATACGTAGAGTGTTAACATG
AAAAAAAACTAAAAATATTACAGGATTTTCTGGCAACGTCTAGGAATAAAATGTTTTTATATAAAATATTGGACGACGAGATACGTAGAGTGTTAACATG
AAAAAATATTACAGGATTTTCTGGCAACGTCTAGAAATAAAATGTTTTTATATAAAATATTGGACGACGAGATACGTAGAGTGTTAACATG
AAAAAAAAAAATATTGGACGACGAGATACGTAGAGTGTTAACATG
AAAAAAAAAATATTGGACGACGAGATACGTAGAGTGTTAACATG
AAAAAAAATATTGGACGACGAGATACGTAGAGTGTTAACATG
AAAAAAATATTGGACGACGAGATACGTAGAGTGTTAACATG
AAAAAATATTGGACGACGAGATACGTAGAGTGTTAACATG
AAAAAATATTGGACGACGAGATACGTAGAGTGTTAACATG
AAAAAATATTGGACGACGAGATACGTAGAGTGTTAACATG
AAAAAATATTGGACGACGAGATACGTAGAGTGTTAACATG
```

E11L ***A1L***

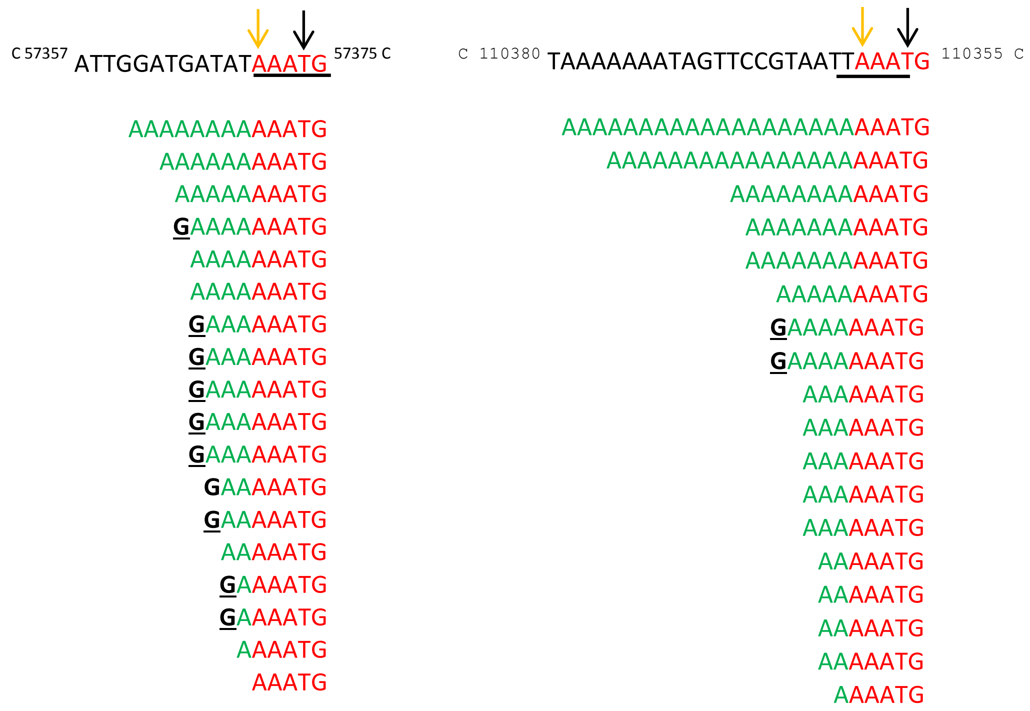

Fig. S2. **5' RACE analysis of selected VACV intermediate transcripts**.
The upper sequences correspond to the viral template DNA with the TSS annotated according to [2] (black arrow) and by us (orange arrow). The INR (underlined) is annotated according to [2]. The sequences depicted below represent individual sequenced cDNA clones. The 5' untranslated regions are shown up to the ATG translation start codon. At least 18 independent cDNA clones were sequenced and aligned with the viral template. The color-coding of the figure and cDNA strand orientation are the same as those in Figure S1.

L3L (WR)

C 79163 TGAGGTTTTCTAGCAGTAGACTCATTTAGAGAAGTTTTTTTTGTGATAAATG 79112 C

AAAAAAAAAAAAAAAAAAAAAAAAAAAAAAAAAAAAAAAAAAAAAAAATG
AAAAAAAAAAAAAAAAAAAAAAAAAAAAAAAAAAAAAAAATG
AAAAAAAAAAAAAAAAAAAAAAAAAAAAAAAATG
AAAAAAAAAAAAAAAAAAAAAAAAAAAAAAAATG
AAAAAAAAAAAAAAAAAAAAAAAAAAAAAAAATG
AAAAAAAAAAAAAAAAAAAAAAAAAAAAAAATG
AAAAAAAAAAAAAAAAAAAAAAAAAAAAAAATG
AAAAAAAAAAAAAAAAAAAAAAAAAAAAAAATG
AAAAAAAAAAAAAAAAAAAAAAAAAAAAAATG
AAAAAAAAAAAAAAAAAAAAAAAAAAAAATG
AAAAAAAAAAAAAAAAAAAAAAAAAAAATG
AAAAAAAAAAAAAAAAAAAAAAAAATG
AAAAAAAAAAAAAAAAAAAAAAATG
AAAAAAAAAAAAAAAAAAAAATG
AAAAAAAAAAAAAAAAAAAATG
AAAAAAAAAAAAAAAAAAATG
AAAAAAAAAAAAAAAATG
AAAAAAAAAAAAAAATG
AAAAAAAAAAAAAATG
AAAAAAAAAAAAAATG
AAAAAAAAAAAAATG
AAAAAAAAAAATG
<u>G</u>AAAAAAAAAATG
AAAAAAAAATG

L3L (vD9muD10mu)

C 79163 TGAGGTTTTCTAGCAGTAGACTCATTTAGAGAAGTTTTTTTTGTGATAAATG 79112 C

AAAAAAAAAAAAAAAAAAAAAAAAAAAAAAAAAAAAAATG
AAAAAAAAAAAAAAAAAAAAAAAAAAAAAAAAAAAAATG
AAAAAAAAAAAAAAAAAAAAAAAAAAAAAAAAATG
AAAAAAAAAAAAAAAAAAAAAAAAAAAAAAATG
AAAAAAAAAAAAAAAAAAAAAAAAAAAAATG
AAAAAAAAAAAAAAAAAAAAAAAAAAAAATG
AAAAAAAAAAAAAAAAAAAAAAAAAAAATG
AAAAAAAAAAAAAAAAAAAAAAAAAAAATG
AAAAAAAAAAAAAAAAAAAAAAATG
AAAAAAAAAAAAAAAAAAAAATG
<u>G</u>AAAAAAAAAAAAAAAAAAAATG
AAAAAAAAAAAAAAAAAAATG
AAAAAAAAAAAAAAAAAAATG
AAAAAAAAAAAAAAAAAAATG
AAAAAAAAAAAAAAAAAAATG
AAAAAAAAAAAAAAAAAAATG
AAAAAAAAAAAAAAAAAAATG
AAAAAAAAAAAAAAAAAATG
AAAAAAAAAAAAAAAAATG
AAAAAAAAAAAAAAAATG
AAAAAAAAAAAAAAAATG
AAAAAAAAAAAAAAATG
AAAAAAAAAAAAATG
AAAAAAAAAAATG
AAAAAAAATG
AAAAAAAATG
AAAAAAAATG

F17R (WR)

```
43659 AAAAATATAGTAGAATTTCATTTTGTTTTTTTCTATGCTATAAATG 43704

AAAAAAAAAAAAAAAAAAAAAAAAAAAAAAAAAAAAAAAATG
AAAAAAAAAAAAAAAAAAAAAAAAAAAAAAAAAAAAAATG
AAAAAAAAAAAAAAAAAAAAAAAAAAAAAAAAAAAAATG
AAAAAAAAAAAAAAAAAAAAAAAAAAAAAAAAAAAATG
AAAAAAAAAAAAAAAAAAAAAAAAAAAAAAAAAAATG
AAAAAAAAAAAAAAAAAAAAAAAAAAAAAAAAAAATG
AAAAAAAAAAAAAAAAAAAAAAAAAAAAAAAAATG
AAAAAAAAAAAAAAAAAAAAAAAAAAAAAAAAATG
AAAAAAAAAAAAAAAAAAAAAAAAAAAAAAAATG
AAAAAAAAAAAAAAAAAAAAAAAAAAAAAAAATG
AAAAAAAAAAAAAAAAAAAAAAAAAAAAAAAATG
AAAAAAAAAAAAAAAAAAAAAAAAAAAAAAAATG
AAAAAAAAAAAAAAAAAAAAAAAAAAAAAAATG
AAAAAAAAAAAAAAAAAAAAAAAAAAAAATG
AAAAAAAAAAAAAAAAAAAAAAAAAAAAATG
AAAAAAAAAAAAAAAAAAAAAAAAAAAATG
AAAAAAAAAAAAAAAAAAAAAAAAAATG
AAAAAAAAAAAAAAAAAAAAAAAAAATG
AAAAAAAAAAAAAAAAAAAAAAAAATG
AAAAAAAAAAAAAAAAAAAAAAATG
AAAAAAAAAAAAAAAAAAAAAATG
AAAAAAAAAAAAAAAATG
GAAAAAAAAAAAAAATG
AAAAAAAAAAAAAATG
AAAAAAAAAAAAAATG
AAAAAAAAAAAATG
```

F17R (vD9muD10mu)

```
43659 AAAAATATAGTAGAATTTCATTTTGTTTTTTTCTATGCTATAAATG 43704

AAAAAAAAAAAAAAAAAAAAAAAAAAAAAAAAAAAAAAAAAATG
AAAAAAAAAAAAAAAAAAAAAAAAAAAAAAAAAAAAAAAATG
AAAAAAAAAAAAAAAAAAAAAAAAAAAAAAAAAATG
AAAAAAAAAAAAAAAAAAAAAAAAAAAAAAAAATG
AAAAAAAAAAAAAAAAAAAAAAAAAAAAAAAAATG
AAAAAAAAAAAAAAAAAAAAAAAAAAAAAAAATG
AAAAAAAAAAAAAAAAAAAAAAAAAAAAAAAATG
AAAAAAAAAAAAAAAAAAAAAAAAAAAAAAATG
AAAAAAAAAAAAAAAAAAAAAAAAAAAATG
AAAAAAAAAAAAAAAAAAAAAAAAAAAATG
AAAAAAAAAAAAAAAAAAAAAAAAAAATG
AAAAAAAAAAAAAAAAAAAAAAAAAAATG
AAAAAAAAAAAAAAAAAAAAAAAAATG
AAAAAAAAAAAAAAAAAAAAAAAAATG
AAAAAAAAAAAAAAAAAAAAAAAATG
AAAAAAAAAAAAAAAAAAAAAAAATG
AAAAAAAAAAAAAAAAAAAAATG
AAAAAAAAAAAAAAAAAAAAATG
AAAAAAAAAAAAAAAAAAATG
AAAAAAAAAAAAAAAAAATG
```

A17L (WR)

C 126228 TTATACTCCATCTTTAATAGTGACATTTTTTAATATATAAATG 126192 C

AAAAAAAAAAAAAAAAAAAAAAAAAAAAAAAAAAATG
AAAAAAAAAAAAAAAAAAAAAAAAAAAAAAAAATG
AAAAAAAAAAAAAAAAAAAAAAAAAAAAAAATG
AAAAAAAAAAAAAAAAAAAAAAAAAAAAAATG
AAAAAAAAAAAAAAAAAAAAAAAAAAATG
AAAAAAAAAAAAAAAAAAAAAAAAAAATG
AAAAAAAAAAAAAAAAAAAAAAAAATG
AAAAAAAAAAAAAAAAAAAAAAAAATG
AAAAAAAAAAAAAAAAAAAAAATG
AAAAAAAAAAAAAAAAAAAATG
AAAAAAAAAAAAAAAAAAATG
AAAAAAAAAAAAAAAAATG
AAAAAAAAAAAAAAAAATG
AAAAAAAAAAAAAAAATG
AAAAAAAAAAAAAAATG
AAAAAAAAAAAAATG
AAAAAAAAAAAAATG
AAAAAAAAAAAAATG

A17L (vD9muD10mu)

C 126228 TTATACTCCATCTTTAATAGTGACATTTTTTAATATATAAATG 126192 C

AAAAAAAAAAAAAAAAAAAAAAAAAAAAAAAAAAAAAATG
AAAAAAAAAAAAAAAAAAAAAAAAAAAAAAAAAAAATG
AAAAAAAAAAAAAAAAAAAAAAAAAAAAAAAAAAATG
AAAAAAAAAAAAAAAAAAAAAAAAAAAAAAAAAATG
AAAAAAAAAAAAAAAAAAAAAAAAAAAAAAAAATG
AAAAAAAAAAAAAAAAAAAAAAAAAAAAAAATG
AAAAAAAGAAAAAAAAAAAAAAAAAAAAAAATG
AAAAAAAAAAAAAAAAAAAAAAAAAAAAAATG
AAAAAAAAAAAAAAAAAAAAAAAAAATG
AAAAAAAAAAAAAAAAAAAAAAAAATG
AAAAAAAAAAAAAAAAAAAAAAAATG
AAAAAAAAAAAAAAAAAAAAAATG
AAAAAAAAAAAAAAAAAAAAAATG
AAAAAAAAAAAAAAAAAAAAATG
AAAAAAAAAAAAAAAAAAAATG
AAAAAAAAAAAAAAAAAAAATG
AAAAAAAAAAAAAAAAAAATG
AAAAAAAAAAAAAAAAAAATG
AAAAAAAAAAAAAAAAATG
AAAAAAAAAAAAAAAAATG
AAAAAAAAAAAAAAAAATG
AAAAAAAAAAAAAATG
GAAAAAAAAAAAAATG
AAAAAAAAAAAAATG
AAAAAAAAAAAATG
GAAAAAAAAAATG

C3L (WR)

C 19505 ACGATGTCCAGGATAACATTTTTACGGATAAATAAATATG 19466 C

AAAAAAAAAAAAAAAAAAAATATG

AAAAAAAAAAAAAAAAAATATG

AAAAAAAAAAAAAAAAAATATG

AAAAAAAAAAAAAAAAATATG

AAAAAAAAAAAAAAAAATATG

AAAAAAAAAAAAAAAAATATG

AAAAAAAAAAAAAAAAATATG

AAAAAAAAAAAAAAAATATG

AAAAAAAAAAAAAAATATG

AAAAAAAAAAAAAAATATG

AAAAAAAAAAAAAATATG

AAAAAAAAAAAATATG

AAAAAAAAAAAATATG

AAAAAAAAAAATATG

AAAAAAAAAAATATG

AAAAAAAAAATATG

AAAAAAAAAATATG

GAAAAAAAAATATG

AAAAAAAAAATATG

AAAAAAAAAATATG

GAAAAAATATG

C3L (vD9muD10mu)

C 19505 ACGATGTCCAGGATAACATTTTTACGGATAAATAAATATG 19466 C

AAAAAAAAAAAAAAAAAAAAAAAATATG

AAAAAAAAAAAAAAAAAAAAAATATG

AAAAAAAAAAAAAAAAAATATG

AAAAAAAAAAAAAAAAAATATG

AAAAAAAAAAAAAAAAAATATG

AAAAAAAAAAAAAAAAATATG

AAAAAAAAAAAAAAAAATATG

AAAAAAAAAAAAAAAAATATG

AAAAAAAAAAAAATATG

AAAAAAAAAAAAATATG

AAAAAAAAAAAATATG

AAAAAAAAAAAATATG

AAAAAAAAAAATATG

AAAAAAAAAAATATG

AAAAAAAAAAATATG

AAAAAAAAAATATG

AAAAAAAAATATG

AAAAAAAAATATG

AAAAAAAAATATG

AAAAAAAATATG

AAAAAAAATATG

GAAAAAAAATATG

GAAAATATG

Fig. S3. 5' RACE analysis of selected VACV late transcripts.

The upper sequences correspond to the viral template DNA with a TSS annotated according to [2] (black arrow) and by us (orange arrow). The INR (underlined) is annotated according to [2]. The sequences depicted below represent individual sequenced cDNA clones. The 5' untranslated regions are shown up to the ATG translation initiation codon. The color-coding of the figure and cDNA strand orientation are the same as those in Figure S1. At least 18 independent clones were sequenced and aligned with the viral DNA.

m^{7}G RNA oligo

1 **[N7-MeGppp]**AAAAAAAAAAAAAAAAATGAGTTATTTAAGATATTACAAT 40

GAAAAAAAAAAAAAAAAAAAAAAAATGAGTTATTTAAGATATTACAAT
GAAAAAAAAAAAAAAAAAAAAAAATGAGTTATTTAAGATATTACAAT
GAAAAAAAAAAAAAAAAAAAAAAATGAGTTATTTAAGATATTACAAT
AAAAAAAAAAAAAAAAAAAAAAATGAGTTATTTAAGATATTACAAT
GAAAAAAAAAAAAAAAAAAAAAATGAGTTATTTAAGATATTACAAT
GAAAAAAAAAAAAAAAAAAAAAATGAGTTATTTAAGATATTACAAT
GAAAAAAAAAAAAAAAAAAAAAATGAGTTATTTAAGATATTACAAT
GAAAAAAAAAAAAAAAAAAAAAATGAGTTATTTAAGATATTACAAT
GAAAAAAAAAAAAAAAAAAAAAATGAGTTATTTAAGATATTACAAT
GAAAAAAAAAAAAAAAAAAAAAATGAGTTATTTAAGATATTACAAT
AAAAAAAAAAAAAAAAAAAAAATGAGTTATTTAAGATATTACAAT
GAAAAAAAAAAAAAAAAAAAAATGAGTTATTTAAGATATTACAAT
GAAAAAAAAAAAAAAAAAAAAATGAGTTATTTAAGATATTACAAT
GAAAAAAAAAAAAAAAAAAAAATGAGTTATTTAAGATATTACAAT
GAAAAAAAAAAAAAAAAAAAAATGAGTTATTTAAGATATTACAAT
GAAAAAAAAAAAAAAAAAAAATGAGTTATTTAAGATATTACAAT
GAAAAAAAAAAAAAAAAAAAATGAGTTATTTAAGATATTACAAT
GAAAAAAAAAAAAAAAAAAAATGAGTTATTTAAGATATTACAAT
AAAAAAAAAAAAAAAAAAAATGAGTTATTTAAGATATTACAAT
AAAAAAAAAAAAAAAAAAAATGAGTTATTTAAGATATTACAAT
AAAAAAAAAAAAAAAAAAATGAGTTATTTAAGATATTACAAT
GAAAAAAAAAAAAAAAAATGAGTTATTTAAGATATTACAAT
GAAAAAAAAAAAAAAAAATGAGTTATTTAAGATATTACAAT

ppp RNA oligo

1 **[ppp]**AAAAAAAAAAAAAAAAATGAGTTATTTAAGATATTACAAT 40

AAAAAAAAAAAAAAAAAAAAAAATGAGTTATTTAAGATATTACAAT
AAAAAAAAAAAAAAAAAAAAAAATGAGTTATTTAAGATATTACAAT
AAAAAAAAAAAAAAAAAAAAAAATGAGTTATTTAAGATATTACAAT
AAAAAAAAAAAAAAAAAAAAAAATGAGTTATTTAAGATATTACAAT
AAAAAAAAAAAAAAAAAAAAAATGAGTTATTTAAGATATTACAAT
AAAAAAAAAAAAAAAAAAAAAATGAGTTATTTAAGATATTACAAT
AAAAAAAAAAAAAAAAAAAAAATGAGTTATTTAAGATATTACAAT
AAAAAAAAAAAAAAAAAAAAAATGAGTTATTTAAGATATTACAAT
AAAAAAAAAAAAAAAAAAAAAATGAGTTATTTAAGATATTACAAT
AAAAAAAAAAAAAAAAAAAAAATGAGTTATTTAAGATATTACAAT
AAAAAAAAAAAAAAAAAAAAATGAGTTATTTAAGATATTACAAT
AAAAAAAAAAAAAAAAAAAAATGAGTTATTTAAGATATTACAAT
AAAAAAAAAAAAAAAAAAAAATGAGTTATTTAAGATATTACAAT
AAAAAAAAAAAAAAAAAAAAATGAGTTATTTAAGATATTACAAT
AAAAAAAAAAAAAAAAAAAAATGAGTTATTTAAGATATTACAAT
AAAAAAAAAAAAAAAAAAAATGAGTTATTTAAGATATTACAAT
AAAAAAAAAAAAAAAAAAAATGAGTTATTTAAGATATTACAAT
AAAAAAAAAAAAAAAAAAAATGAGTTATTTAAGATATTACAAT
AAAAAAAAAAAAAAAAAAAATGAGTTATTTAAGATATTACAAT
AAAAAAAAAAAAAAAAAAAATGAGTTATTTAAGATATTACAAT
AAAAAAAAAAAAAAAAAAATGAGTTATTTAAGATATTACAAT
AAAAAAAAAAAAAAAAAAATGAGTTATTTAAGATATTACAAT
AAAAAAAAAAAAAAAAAATGAGTTATTTAAGATATTACAAT
AAAAAAAAAAAAAAAAATGAGTTATTTAAGATATTACAAT
AAAAAAAAAAAAAAAAATGAGTTATTTAAGATATTACAAT

Fig. S4. **5' RACE analysis of control RNA oligonucleotides.** The upper sequences correspond to the designed and ordered sequence. The sequences depicted below represent individual sequenced cDNA clones. The color-coding of the figure is the same as those in Figure S1.

A17L **(RLM-RACE)**

C 126228 TTATACTCCATCTTTAATAGTGACATTTTTTAATATATAAATG 126192 C

AAAAAAAAAAAAAAAAAAAAAAAAAAAAAAAATG
AAAAAAAAAAAAAAAAAAAAAAAAAAAAATG
AAAAAAAAAAAAAAAAAAAAAAAAAAAATG
AAAAAAAAAAAAAAAAAAAAAAAAAAATG
AAAAAAAAAAAAAAAAAAAAAAAAAATG
AAAAAAAAAAAAAAAAAAAAAAAAAATG
AAAAAAAAAAAAAAAAAAAAAAAAAATG
AAAAAAAAAAAAAAAAAAAAAAAAAATG
AAAAAAAAAAAAAAAAAAAAAAAAATG
AAAAAAAAAAAAAAAAAAAAAAAAATG
AAAAAAAAAAAAAAAAAAAAAAAATG
AAAAAAAAAAAAAAAAAAAAAAAATG
AAAAAAAAAAAAAAAAAAAAAAAATG
AAAAAAAAAAAAAAAAAAAATG
AAAAAAAAAAAAAAAAATG
AAAAAAAAAAAAAATG
AAAAAAAAAAAAATG
AAAAAAAAAAAAATG
AAAAAAAAAAAATG
AAAAAAAAAAAATG
AAAAAAAAAAATG
AAAAAAAAAAATG
AAAAAAAAATG
AAAAAAAAATG
AAAAAAAATG
AAAAAATG
AAAAAATG
AAAAAATG
AAAATG

C3L **(RLM-RACE)**

C 19505 ACGATGTCCAGGATAACATTTTTACGGATAAATAAATATG 19466 C

AAAAAAAAAAAAAAAAAAAAAAAAATATG
AAAAAAAAAAAAAAAAAAAAAAATATG
AAAAAAAAAAAAAAAAAAAAAAATATG
AAAAAAAAAAAAAAAAAAAAAAATATG
AAAAAAAAAAAAAAAAAAAAAATATG
AAAAAAAAAAAAAAAAAAAAATATG
AAAAAAAAAATATG
AAAAAAAAAATATG
AAAAAAAAATATG
AAAAATATG
AAAAATATG
AAAAATATG
AAAAATATG
AAAAATATG
AAAAATATG
AAAAATATG
AAAAATATG
AAATATG
AAATATG
AAATATG
AAATATG
AAATATG
AAATATG
AAATATG
AAATATG
AAATATG
AAATATG
AAATATG

Fig. S5. 5' RLM-RACE analysis of the VACV *A17L* and *C3L* late transcripts. INRs (underlined) were annotated within the upper sequences of the viral template DNA according to [2]. The sequences below represent individual sequenced cDNA clones obtained by the 5' RLM-RACE method (oligocapping). The 5' untranslated regions are shown in full up to the ATG translation start codon. At least 18 independent cDNA clones were sequenced and aligned with the viral template DNA. Sequence parts that share nucleotide

identity with the template viral DNA are labeled in red, and sequence parts that are not identical to the template viral DNA are labeled in green.

Gene	Primer name	Sequence (5'-3')	Used in
A1L	5RACE_VV_A1L	CGTAAACGCCGTCTTTATCTC	5' RACE
A2L	5RACE_VV_A2L	GAAGGAGTGTTTTCTTGGCAA	5' RACE / 5' end assay
	A2L_Forw	CGATTATGTAGCGGTTGTAGAC	5' end assay
A5R	5RACE_VV_A5R	AGAACCCTCCTCTATCTCTTG	5' RACE
A17L	5RACE_VV_A17L	CTTCTATAGTCCTGTCTTTCG	5' RACE / oligo-capping
C3L	5RACE_VV_C3L	CCTCGGGATTCCATACCATAG	5' RACE / oligo-capping / 5' end assay
	C3L_Forw	ATGAAGGTGGAGAGCGTG	5' end assay
D5R	5RACE_VV_D5R	ATCCATAGTGGTATACGTGTC	5' RACE
D12L	5RACE_VV_D12L	CACGTCGAAGGTTAACATCTT	5' RACE
E11L	5RACE_VV_E11L	CACGACTGAAATAACCGCGTG	5' RACE
F17R	5RACE_VV_F17R	TAGTTCAAAAGTCTCGACGCC	5' RACE
G8R	5RACE_VV_G8R	GAATGACGGTTCTACCACAAC	5' RACE
	VV_G8Rp_1	TCGACCATTTAACTTTAAATAATTTACAAAAATTTAAAATG	*pG8R^{N}*-EGFP reporter vector construction
	VV_G8Rp_2	GATCCATTTTAAATTTTTGTAAATTATTTAAAGTTAAATGG	
G8R^{PM}	VV_G8Rp_mut_1	TCGACCATTTAACTTTAAATAATTTACAAAAATTTAACATG	*pG8R^{PM}*-EGFP reporter vector construction
	VV_G8Rp_mut_2	GATCCATGTTAAATTTTTGTAAATTATTTAAAGTTAAATGG	
H5R	5RACE_VV_H5R	TTACCAGCTTCAACTTGTACC	5' RACE
I4L	5RACE_VV_I4L	GGTCTTTCAACGATCTTGTTG	5' RACE
J6R	5RACE_VV_J6R	GGTTGCATACATTCACTGTTC	5' RACE / 5' end assay
	J6R_Forw	GGCTGTAATCTCTAAGGTTACG	5' end assay
K1L	5RACE_VV_K1L	CATCATTAAGCATGACGGCAT	5' RACE
L3L	5RACE_VV_L3L	ACGTTTAGCCGCCTTTAATAG	5' RACE
RNA oligo	ppp-RNA	[ppp]AAAAAAAAAAAAAAAAUGAGUUAUUUAAGAUAUUACAAU	control RNA oligo analyses
	m7G-RNA	[N7MeGppp]AAAAAAAAAAAAAAAAUGAGUUAUUUAAGAUAUUACAAU	
	RT_for_RNA_olig	ATTGTAATATCTTAAATAAC	
	RNA_oligo_nested_I	CATCAGGCAGTTTAAGTCGGGACAATAGGAGCCGCAATACACAGTTTACCGCATCTTGACCTAACTGACATACTGCCAATTGTAATATCTTAAATAAC	
universal	olig2(dC)anchor	GACCACGCGTATCGATGTCGACCCCCCCCCCCC	5' RACE

universal	5' RACE Adapter	GCUGAUGGCGAUGAAUGAACACUGCGUUUGCUGGCUUUGAUGAAA	oligo-capping
universal	5' RACE Outer Primer	GCTGATGGCGATGAATGAACACTG	oligo-capping
universal	5' RACE Inner Primer	CGCGGATCCGAACACTGCGTTTGCTGGCTTTGATG	oligo-capping

Table S1. Oligonucleotides used in this study.

Summary of the 5' RACE experiments done in HeLa cells

Vaccinia gene time class	Gene	INR	Number of 5' nontemplated adenosines	Minimal number of adenosines[1] at 5' ends of uncapped transcripts	mRNAs with 5' nontemplated poly(A) leaders [%]	Median/Mean of added adenosines per mRNA molecule	5' capped mRNAs [%]	Number of analyzed clones
Early	***J6R***	no	0	0	0	0/0	100	20
	H5R	no	0	0	0	0/0	100	20
	K1L	no	0	0	0	0/0	100	25
	I4L	no	0	0	0	0/0	100	23
	A5R	yes	0 – 7	4	64.7	1/1.8	58.8	17
	D12L	yes	0 – 5	3	73.3	3/2.3	40.0	15
	D5R	yes	1- 11	5	100	4 / 4.1	57.1	21
Intermediate	***G8R***	yes	0 – 15	6	88.9	3/3.9	33.3	18
	A1L	yes	1 – 19	4	100	3/5.2	11.1	18
	A2L	yes	1 - 8	6	100	3 / 3.5	36.4	22
	E11L	yes	0 - 8	3	94.4	3 / 3.1	55.6	18
Late	***C3L***	yes	3 – 17	9	100	11/10.5	9.52	21
	A17L	yes	9 – 32	12	100	17/18.3	0	18
	L3L	yes	6 - 44	9	100	21/20.9	4.1	24
	F17R	yes	8 - 37	11	100	26/24	3.9	26

[1] templated and nontemplated

Summary of the 5' RACE experiments done in BHK-21 cells

Vaccinia gene time class	Gene	INR	Number of 5' nontemplated adenosines	Minimal number of adenosines[1] at 5' ends of uncapped transcripts	mRNAs with 5' nontemplated poly(A) leaders [%]	Median/Mean of added adenosines per mRNA molecule	5' capped mRNAs [%]	Number of analyzed clones
Early	***K1L***	no	0	0	0	0/0	100	25
	J6R	yes	0 - 6	5	85	2/2.3	20	20
Intermediate	***G8R***	yes	0 - 14	5	96.1	5.5/5	19.2	22
	A2L	yes	1 - 7	6	100	3/3	0	22
Late	***C3L***	yes	1 - 21	8	100	9/10	8.7	23
	A17L	yes	8 - 35	11	100	18/19.9	7.7	26
	L3L	yes	5 - 34	8	100	16/17.7	3.7	27
	F17R	yes	18 - 39	18	100	24.5/25.2	0	20

[1] templated and nontemplated

Table S2. Summary of the 5' RACE experiments done in HeLa and BHK-21 cells. Each VACV gene is characterized by the gene name; presence or absence of the INR; number of adenosines added in a nontemplated manner; minimal number of total adenosines (both templated and nontemplated) at the 5' ends of uncapped transcripts; percentage of all transcripts containing 5' nontemplated adenosine nucleotides; the average number (shown as the median and mean) of nontemplated adenosine nucleotides per mRNA molecule; percentage of all transcripts containing a 5' cap; and number of analyzed cDNA clones. Related to Figures 1, 2, 4, 8.

Gene time class	Number of clones	5' mRNA cap occurrence [%]	Median/Mean of total length of 5' poly(A) leader [nts]
**early w/o INR**	88	100	-/-
**early with INR**	53	53	6/6
**intermediate**	76	34	7/7
**late**	89	4	22/20

Table S3. Occurrence of 5' mRNA cap structures and length of the 5' poly(A) leader in VACV transcripts from different GTCs. Related to Figure 3.

Number of nontemplated adenosines	Number of sequences		Number of sequences with m^7G structure		5' mRNA cap occurrence [%]	
	HeLa	BHK-21	HeLa	BHK-21	HeLa	BHK-21
**0**	101	27	93	27	92	100
**1**	18	11	9	4	50	36
**2**	22	12	6	1	27	8.3
**3**	27	19	14	1	52	5.2
**4**	26	4	8	1	31	25
**5**	6	9	0	0	0	0
**6+**	106	103	4	5	4	4.8

Table S4. Proportion of mRNAs containing 5' cap structures among VACV mRNAs with different numbers of nontemplated adenosines in their 5' poly(A) leaders. Related to Figure 4.

RNA oligo name	Sequence (5' → 3')	Number of added adenosines	RNA oligos with 5' added poly(A) [%]	Median / Mean of added adenosines per RNA oligo	5' capped RNA oligos [%]	Number of analyzed clones
ppp-RNA	[ppp]AAAAAAAAAAAAAAAAAUGAGUU AUUUAAGAUAUUACAAU	0-6	92.3	4/3.81	0	25
m7G-RNA	[N^7MeGppp]AAAAAAAAAAAAAAAAAU GAGUUAUUUAAGAUAUUACAAU	0-7	91.3	4/4.04	78.3	23

Table S5. Analysis of control RNA oligonucleotides. Each RNA oligonucleotide is characterized by the name; number of added adenosines; percentage of oligonucleotides containing 5' added adenosine nucleotides; the average number (shown as the median and mean) of added adenosine nucleotides per RNA oligonucleotide; percentage of RNA oligonucleotides containing a 5' cap; and number of analyzed cDNA clones. Related to Figures 2, S4.

Vaccinia gene time class	Method	Gene	Number of 5' nontemplated adenosines	mRNAs with 5' nontemplated poly(A) leaders [%]	Median/Mean of added adenosines per mRNA molecule	5' capped mRNAs [%]	Number of analyzed clones
Late	5' RACE	*C3L**	9–17	100	11/10.5	9.5	21
		*A17*L*	9–32	100	17/18.3	0	18
Late	RLM-RACE	*C3L*	0–21	60.7	2/5.2	N/A	28
		A17L	1–28	100	14/14.2	N/A	29

[1] templated and nontemplated
* data from Table S2a

Table S6. **5' RLM-RACE analysis of VACV *A17L* and *C3L* late transcripts**. The results of 5' RLM-RACE in combination with the results of classic 5' RACE-PCR (data from Table S2) show that transcripts of both selected VACV late genes containing the 5' mRNA cap structure have shorter 5' poly(A) leaders than uncapped transcripts of the same genes. Related to Figure 6.

Vaccinia gene time class	Promoter	Number of 5' nontemplated adenosines	Minimal number of adenosines[1] at 5' end of uncapped transcripts	mRNAs with 5' nontemplated poly(A) leaders [%]	Median/Mean of added adenosines per mRNA molecule	5' capped mRNAs [%]	Number of analyzed clones
Intermediate	***G8R*[N]***	6–15	6	88.9	3/3.9	33.3	18
	***G8R*[P]**	0–9	3	83.3	2.5/3	20.8	24
	***G8R*[PM]**	0–1	2	31.6	0/0.3	57.9	19

[1] templated and nontemplated

[N]* data from Table S2, native *G8R* promoter, VACV mRNA purified from infected HeLa cells

[P] plasmid-localized reporter gene (*EGFP*) under the control of the *G8R* VACV intermediate promoter

[PM] plasmid-localized reporter gene (*EGFP*) under the control of the *G8R* VACV intermediate promoter containing a single point mutation in the INR

Table S7. VACV INR controls 5' end formation of viral mRNAs. The results show that a single A/C substitution within the short VACV INR leads to a significant reduction in mRNA 5' poly(A) leader length and to the switching of mRNA ends from 5' polyadenylated to 5' capped. Related to Figure 7.

	G8R^{N}*****	***G8R***^{P}
G8R^{P}	0.764956	
G8R^{PM}	0.000037	0.000037

* native *G8R* promoter, VACV mRNA purified from infected HeLa cells
^{P} plasmid localized reporter gene (*EGFP*) under the control of a *G8R* VACV intermediate promoter
^{PM} plasmid localized reporter gene (*EGFP*) under the control of the *G8R* VACV intermediate promoter containing a single point mutation in the INR

Table S8. The results of the statistical analysis are depicted in Figure 7. *Dunn p*-values, further adjusted by the *Benjamini-Hochberg FDR* method, are depicted in the table above. Related to Figure 7.

	early	intermediate
**intermediate**	5.220964e-02	
**late**	1.508969e-25	3.257361e-21

Table S9. **The results of the statistical analysis are depicted in Figure 3.** *Dunn p*-values, further adjusted by the *Benjamini-Hochberg FDR* method, are depicted in the table above.

Supplementary references

1 Yang, Z., Bruno, D.P., Martens, C.A., Porcella, S.F., and Moss, B. (2011). Genome-wide analysis of the 5' and 3' ends of vaccinia virus early mRNAs delineates regulatory sequences of annotated and anomalous transcripts. J Virol *85*, 5897-5909.

2 Yang, Z., Martens, C.A., Bruno, D.P., Porcella, S.F., and Moss, B. (2012). Pervasive initiation and 3'-end formation of poxvirus postreplicative RNAs. J Biol Chem *287*, 31050-31060.
