# Supplementary material for: Vaccinia virus mRNAs containing long 5′-poly(A)-leaders lack a canonical 5′-methylguanosine cap
Source: Nat Commun. 2025 Dec 22;16:11340. doi: 10.1038/s41467-025-67916-w (PMC12722222; doi:10.1038/s41467-025-67916-w)
Supplement: Supplementary file 2 — Reporting Summary [file 41467_2025_67916_MOESM2_ESM.pdf]

nature portfolio

Corresponding author(s): Martin Pospisek

Last updated by author(s): Dec 3, 2025

Reporting Summary

Nature Portfolio wishes to improve the reproducibility of the work that we publish. This form provides structure for consistency and transparency in reporting. For further information on Nature Portfolio policies, see our Editorial Policies and the Editorial Policy Checklist.

Statistics

For all statistical analyses, confirm that the following items are present in the figure legend, table legend, main text, or Methods section.

n/a	Confirmed	
☐	☒	The exact sample size (*n*) for each experimental group/condition, given as a discrete number and unit of measurement
☐	☒	A statement on whether measurements were taken from distinct samples or whether the same sample was measured repeatedly
☐	☒	The statistical test(s) used AND whether they are one- or two-sided<br>*Only common tests should be described solely by name; describe more complex techniques in the Methods section.*
☐	☒	A description of all covariates tested
☐	☒	A description of any assumptions or corrections, such as tests of normality and adjustment for multiple comparisons
☐	☒	A full description of the statistical parameters including central tendency (e.g. means) or other basic estimates (e.g. regression coefficient) AND variation (e.g. standard deviation) or associated estimates of uncertainty (e.g. confidence intervals)
☐	☒	For null hypothesis testing, the test statistic (e.g. *F*, *t*, *r*) with confidence intervals, effect sizes, degrees of freedom and *P* value noted<br>*Give P values as exact values whenever suitable.*
☒	☐	For Bayesian analysis, information on the choice of priors and Markov chain Monte Carlo settings
☒	☐	For hierarchical and complex designs, identification of the appropriate level for tests and full reporting of outcomes
☐	☒	Estimates of effect sizes (e.g. Cohen's *d*, Pearson's *r*), indicating how they were calculated

Our web collection on statistics for biologists contains articles on many of the points above.

Software and code

Policy information about availability of computer code

Data collection	*Provide a description of all commercial, open source and custom code used to collect the data in this study, specifying the version used OR state that no software was used.*
Data analysis	Microsoft Excel 365, Origin, https://astatsa.com/, G*Power 3

For manuscripts utilizing custom algorithms or software that are central to the research but not yet described in published literature, software must be made available to editors and reviewers. We strongly encourage code deposition in a community repository (e.g. GitHub). See the Nature Portfolio guidelines for submitting code & software for further information.

Data

Policy information about availability of data

All manuscripts must include a data availability statement. This statement should provide the following information, where applicable:

- Accession codes, unique identifiers, or web links for publicly available datasets
- A description of any restrictions on data availability
- For clinical datasets or third party data, please ensure that the statement adheres to our policy

The experimental data including sequences are included in the manuscript and supplementary file.

Research involving human participants, their data, or biological material

Policy information about studies with human participants or human data. See also policy information about sex, gender (identity/presentation), and sexual orientation and race, ethnicity and racism.

Reporting on sex and gender	N/A
Reporting on race, ethnicity, or other socially relevant groupings	N/A
Population characteristics	N/A
Recruitment	N/A
Ethics oversight	N/A

Note that full information on the approval of the study protocol must also be provided in the manuscript.

Field-specific reporting

Please select the one below that is the best fit for your research. If you are not sure, read the appropriate sections before making your selection.

☒ Life sciences ☐ Behavioural & social sciences ☐ Ecological, evolutionary & environmental sciences

For a reference copy of the document with all sections, see nature.com/documents/nr-reporting-summary-flat.pdf

Life sciences study design

All studies must disclose on these points even when the disclosure is negative.

Sample size	Statistical analyses were performed exactly according to Vopalensky et al., Front Microbiol. 2019 Oct 30;10:2366. Briefly, the variance in the 5' poly(A) leader lengths of selected VACV mRNAs was analyzed using the nonparametric Kruskal-Wallis test followed by the post hoc Dunn test with p-value adjustment according to the Benjamini-Hochberg FDR method. These data did not follow a normal distribution according to the Shapiro-Wilk test. Categorical binary data of 5' mRNA cap and 5' poly(A) occurrence in VACV transcripts were evaluated using two-tailed Fisher's exact test with the 95% confidence interval calculated using the adjusted Wald method. Sample size calculations were performed with G*Power 3 software (Faul et al.,2007).
Data exclusions	We did not exclude any data.
Replication	All the replications are clearly described and visible in the manuscript and supplementary file. A total of 491 unbiased cDNA clones were sequenced and analyzed further.
Randomization	N/A
Blinding	N/A

Reporting for specific materials, systems and methods

We require information from authors about some types of materials, experimental systems and methods used in many studies. Here, indicate whether each material, system or method listed is relevant to your study. If you are not sure if a list item applies to your research, read the appropriate section before selecting a response.

Materials & experimental systems

n/a	Involved in the study	
☒	☐	Antibodies
☐	☒	Eukaryotic cell lines
☒	☐	Palaeontology and archaeology
☒	☐	Animals and other organisms
☒	☐	Clinical data
☒	☐	Dual use research of concern
☒	☐	Plants

Methods

n/a	Involved in the study	
☒	☐	ChIP-seq
☒	☐	Flow cytometry
☒	☐	MRI-based neuroimaging

Eukaryotic cell lines

Policy information about cell lines and Sex and Gender in Research

Cell line source(s)	Epithelial cell lines HeLa (human cervical carcinoma), BSC-40 (African green monkey kidney cells) and BHK-21 (Baby hamster kidney-21 cells) were from the Melkova's laboratory.
Authentication	We haven't authenticate the cell lines prior the experiments. The cell lines were used for virus replication.
Mycoplasma contamination	Mycoplasma tests were regularly performed and were negative.
Commonly misidentified lines (See ICLAC register)	N/A

Plants

Seed stocks	N/A
Novel plant genotypes	N/A
Authentication	N/A
